# The Influence of Facial Signals on the Automatic Imitation of Hand Actions

**DOI:** 10.3389/fpsyg.2016.01653

**Published:** 2016-10-26

**Authors:** Emily E. Butler, Robert Ward, Richard Ramsey

**Affiliations:** School of Psychology, Wales Institute for Cognitive Neuroscience, Bangor UniversityBangor, UK

**Keywords:** imitation, automatic, face signals, social cognition

## Abstract

Imitation and facial signals are fundamental social cues that guide interactions with others, but little is known regarding the relationship between these behaviors. It is clear that during expression detection, we imitate observed expressions by engaging similar facial muscles. It is proposed that a cognitive system, which matches observed and performed actions, controls imitation and contributes to emotion understanding. However, there is little known regarding the consequences of recognizing affective states for other forms of imitation, which are not inherently tied to the observed emotion. The current study investigated the hypothesis that facial cue valence would modulate automatic imitation of hand actions. To test this hypothesis, we paired different types of facial cue with an automatic imitation task. Experiments 1 and 2 demonstrated that a smile prompted greater automatic imitation than angry and neutral expressions. Additionally, a meta-analysis of this and previous studies suggests that both happy and angry expressions increase imitation compared to neutral expressions. By contrast, Experiments 3 and 4 demonstrated that invariant facial cues, which signal trait-levels of agreeableness, had no impact on imitation. Despite readily identifying trait-based facial signals, levels of agreeableness did not differentially modulate automatic imitation. Further, a Bayesian analysis showed that the null effect was between 2 and 5 times more likely than the experimental effect. Therefore, we show that imitation systems are more sensitive to prosocial facial signals that indicate “in the moment” states than enduring traits. These data support the view that a smile primes multiple forms of imitation including the copying actions that are not inherently affective. The influence of expression detection on wider forms of imitation may contribute to facilitating interactions between individuals, such as building rapport and affiliation.

## Introduction

Imitation and facial signals are key social cues that help guide behavior. Imitation between interaction partners increases affiliation and rapport (Chartrand and Van Baaren, 2009; van Baaren et al., 2009), whilst information from faces can convey what someone thinks, feels and desires (Haxby et al., 2000; Blakemore et al., 2004). Recent research has started to identify social antecedents to imitation, including a desire to affiliate and pre-existing rapport (Heyes, 2011; Chartrand and Lakin, 2013). However, less research has shown how facial signals regulate imitative behavior. The aim of the current study is to estimate the impact of recognizing different types of facial signal on imitative behavior.

Faces signal a vast array of social information, which can be used to infer intentions and predict behavior (Haxby et al., 2000; Blakemore et al., 2004). Changeable aspects of faces, such as frowning and smiling expressions, convey emotional states, such as anger and happiness (Tottenham et al., 2009). By contrast, invariant facial features, such as jaw shape or skin tone, are perceived as signaling stable personality features. For example, invariant facial features have been found to indicate trait levels of trustworthiness and dominance (Todorov et al., 2008, 2015), as well as extraversion and agreeableness (Penton-Voak et al., 2006; Kramer and Ward, 2010). As such, faces signal information about a person's current state as well as enduring trait characteristics, both of which influence the regulation of social interactions (Frith, 2009).

The cognitive and neurobiological mechanisms that underpin perception and detection of facial signals have been studied extensively (Haxby et al., 2000; Kanwisher, 2010; Said et al., 2011). With respect to expression perception, motor theories suggest that facial expressions are represented, at least partly, within motor structures of the observer's brain. It is claimed that motor system engagement reflects a process of simulating or automatically imitating observed facial expressions, which contributes to understanding another's emotional state (Niedenthal et al., 2001; Goldman and Sripada, 2005; Moody et al., 2007; Oberman et al., 2007; Sato et al., 2013; Rychlowska et al., 2014; Wood et al., 2016). A key focus of prior research, therefore, has been to investigate the role of facial imitation in the recognition of emotional states.

The influence of affective and other social signals on imitation is not restricted to the response of facial muscles, however. Social signals have also been shown to facilitate and inhibit imitative responses that extend beyond facial imitation to include the copying of bodily postures and gestures (for a review, see Chartrand and Lakin, 2013). For example, facial attractiveness (Van Leeuwen et al., 2009), stigmatized features such as facial scars and obesity (Johnston, 2002), group membership (Yabar et al., 2006), social status (Cheng and Chartrand, 2003), and social exclusion (Lakin et al., 2008) have also been shown to up- or down-regulate imitation. In these studies, participants take part in a task with a confederate, which is completely incidental to the measure of imitation (e.g., describe photographs to a partner). Unbeknownst to the participant, the interaction with the confederate is recorded and the frequency of copying behaviors is used as a measure of how much participants tended to spontaneously copy the confederates actions, such as nose scratches or leg movements. By measuring live social interactions, these studies have high ecological validity but suffer limitations upon experimental control, which makes it difficult to manipulate facial emotions as well as other facial cues. As a consequence, it remains poorly understood how expressions and other facial signals impact imitation of non-facial movements, which are not intrinsically linked to the affective state observed.

The desire to affiliate or disaffiliate has been proposed as a critical factor that regulates imitation (Chartrand and Lakin, 2013). Cues from the face can signal affiliative motivations (Bourgeois and Hess, 2008) and therefore appear good candidates for regulating imitation (Rauchbauer et al., 2015). To date, only four studies have investigated how facial signals impact the imitation of movements that are not controlled by facial muscles and have little to no inherent affective value (Crescentini et al., 2011; Wang et al., 2011; Grecucci et al., 2013; Rauchbauer et al., 2015). These studies used a lab-based reaction time (RT) measure of imitation, which involves making a finger movement, whilst simultaneously observing the finger movement of another person (Brass et al., 2000). The observed finger movement can either be congruent or incongruent with the intended movement. Interference to RTs is measured by longer reaction times when participants observe a finger movement that is incongruent compared to congruent with their intended movement. It is suggested that the interference to RT, in part, represents the cost of inhibiting an imitative response (Brass and Heyes, 2005; Heyes, 2011).

Of the four studies to date, one has shown that direct compared to averted eye-gaze produced greater interference on the imitation task (Wang et al., 2011). Wang et al. (2011) showed that RTs were faster on congruent trials for direct than averted eye gaze, with a smaller and less reliable influence observed on incongruent trials. The influence of eye gaze on congruent trials suggests that direct eye-gaze has a facilitatory effect on imitative responses (Wang et al., 2011). Moreover, the effects of eye-gaze on imitation appear to be robust since they were replicated in a second experiment and the effects were medium or large in magnitude according to Cohen's benchmarks^1^ (Cohen, 1992; Table 1). As such, these findings may suggest that eye-gaze provides an affiliative signal, which enhances imitation (Wang et al., 2011).

**Table 1:** **Results from prior behavioral studies investigating the influence of facial signals on a reaction time measure of automatic imitation**.

**Study/Sample size/Manipulation**	**Contrast**	**Mean difference (ms)**		**95% confidence intervals**	**Cohen's d_z_**	**Bayes factor BF_01_**
Crescentini et al., 2011	Angry > Sad					
*N* = 19		CE	−17.96	[−48.42, 12.50]	−0.28	2.18
Facial expressions		Congruent	1.06	[−16.31, 18.42]	0.03	4.18
		Incongruent	−16.91	[−40.65, 6.84]	−0.34	1.64
	Angry > Neutral					
		CE	3.62	[−25.88, 33.12]	0.06	4.09
		Congruent	−11.45	[−35.39, 12.50]	−0.23	2.72
		Incongruent	−7.83	[−32.16, 16.51]	−0.15	3.44
	Sad > Neutral					
		CE	21.58	[−9.58, 52.74]	0.33	1.72
		Congruent	−12.50	[−29.74, 4.74]	−0.23	1.59
		Incongruent	9.08	[−15.82, 33.98]	0.17	3.25
Grecucci et al., 2013	Fear > neutral					
*N* = 15 (typical individuals)		CE	−14.55	[−74.68, 45.59]	−0.13	3.39
		Congruent	−31.64	[−61.33, −1.95]	−0.59	0.54
Facial expressions		Incongruent	−46.19	[−94.56, 2.18]	−0.53	0.76
Rauchbauer et al., 2015	Happy > Angry					
*N* = 27		CE	13.27	[5.69, 20.84]	0.69	0.04
Facial expressions		Congruent	−8.87	[−14.32, −3.42]	−0.65	0.06
		Incongruent	4.39	[−2.72, 11.50]	0.25	2.38
Wang et al., 2011	Direct > Averted eye gaze					
Exp. 1 *N* = 20		CE	10.71	[3.75, 17.67]	0.72	0.1
Eye gaze		Congruent	−16.16	[−20.93, −11.38]	−1.58	< 0.01
		Incongruent	5.45	[−1.15, 12.04]	0.39	1.23
	Direct > Averted eye gaze					
Exp. 2 *N* = 23		CE	11.00	[4.07, 17.92]	0.69	0.08
Eye gaze		Congruent	−14.65	[−19.93, −9.36]	−1.20	< 0.01
		Incongruent	3.65	[−1.74, 9.04]	0.29	1.93
Flashing box	Center > Side Flash					
		CE	−2.04	[−12.15, 8.08]	−0.09	4.23
		Congruent	−1.21	[−5.95, 3.52]	−0.11	4.02
		Incongruent	3.25	[−3.74, 10.23]	−.2	3.02

*Authors from each study were contacted and kindly provided the raw data, which these analyses are based on*.

Three further studies investigated the impact of facial expressions on automatic imitation (Crescentini et al., 2011; Grecucci et al., 2013; Rauchbauer et al., 2015). Two studies failed to show that sad, angry, or fearful facial expressions had an impact on imitation compared to neutral expressions (Crescentini et al., 2011; Grecucci et al., 2013). A further study showed that happy expressions increased the tendency to imitate compared to angry expressions (Rauchbauer et al., 2015). The previous studies address additional questions regarding the effect of group membership on imitation (Rauchbauer et al., 2015) and the influence of facial expression on imitation in atypical groups (Grecucci et al., 2013), as well as two being fMRI studies (Crescentini et al., 2011; Rauchbauer et al., 2015). However, the current review of their findings will focus on those most relevant to the current study, those pertaining to the behavioral effect of perception of facial expression on imitation in typical individuals. Rauchbauer et al. (2015) showed that the congruency effect (the difference in RTs between incongruent and congruent trials) was greater following happy than angry expressions and the effect size for this difference was medium-to-large in magnitude (Table 1). Further, the effect was largely driven by differences on congruent trials with a smaller and less consistent effect observed on incongruent trials. The authors interpret the result to suggest that in response to an affiliative signal (a smile), greater imitation reflects the desire to reciprocate the affiliative signal and thus build rapport with an interaction partner. As summarized in Table 1, only a small number of studies to date have investigated the role of facial signals on the automatic imitation of hand actions and the most convincing evidence to date shows that imitation is sensitive to direct more than averted eye contact (Wang et al., 2011) and happy compared to angry expressions (Rauchbauer et al., 2015).

In sum, although many studies have investigated the role of facial imitation during the recognition of expressions (Niedenthal et al., 2001), there is little known regarding the consequences of recognizing affective states for other forms of imitation. For instance, there are few estimates of how facial expressions impact imitation of non-facial movements, which are not intrinsically linked to the affective state observed. In addition, no study to date has investigated how stable trait information cued by the face influences imitation. Indeed, trait cues to agreeableness can be readily perceived from the face and indicate whether to approach or avoid someone (Penton-Voak et al., 2006; Todorov et al., 2008; Kramer and Ward, 2010), but it remains unknown how such signals influence imitation.

The aim of the current study is to further investigate the role of facial signals in regulating imitation. First, we aim to clarify the influence that expressions have in regulating imitation by incorporating positive, negative, and neutral facial signals in the same experiment, thus building on prior studies (Crescentini et al., 2011; Grecucci et al., 2013; Rauchbauer et al., 2015). Given low levels of reproducibility in psychology and the importance of attempts to replicate effects (Simmons et al., 2011; Cumming, 2012; Open Science Collaboration, 2015), we will provide a further estimate of the extent to which happy expressions lead to greater imitation than angry expressions (Rauchbauer et al., 2015). Second, to our knowledge, we will provide the first estimate of how invariant features of the face, which cue trait-level agreeableness, influence imitation. Across a series of experiments, faces that signal emotional states as well as enduring traits will be presented before and during the completion of an automatic imitation task (Brass et al., 2000). Experiments 1 and 2 will investigate the extent to which facial expressions impact automatic imitation, whereas Experiments 3 and 4 will investigate the extent to which trait information from faces influences automatic imitation. The current study will be able to reveal similarities and differences between the way imitative mechanisms operate following the detection of transient expressions and stable trait information from faces.

## Experiment 1

### Introduction

To date, one study has shown that happy expressions increase the tendency to imitate compared to angry faces (Rauchbauer et al., 2015) and two studies have failed to show that detecting negative facial expressions has any impact on automatic imitation (Crescentini et al., 2011; Grecucci et al., 2013). Across two experiments, we aim to clarify the influence of emotional expressions on imitation by comparing the influence of positive, negative, and neutral facial expressions. Experiment 1 investigates the extent to which happy and angry facial expressions influence automatic imitation compared with a neutral facial expression.

### Methods

#### Participants

Twenty-eight Caucasian Bangor University students participated for course credit. Throughout all experiments, participants were removed from analyses if mean accuracy or mean RT for congruent or incongruent trials was greater than three standard deviations (>3SD) from the group mean. One participant was removed from the automatic imitation part of the experiment due to equipment failure; however, their data is included in the ratings part of the experiment. Of the remaining 27 (22 female, 5 male; *M*_age_ = 19.33 years, *SD* = 1.33) all had normal or corrected-to-normal vision and 25 were right handed as measured by the Edinburgh Handedness Inventory. This measure showed that two participants were ambidextrous, although they were included in the sample as they reported being predominantly right-hand users. The study complied with the guidelines set by the Research Ethics and Governance Committee of the School of Psychology at Bangor University.

#### Task and stimuli

##### Face evaluation task

Stimuli were images of 12 individuals from the NimStim data set (models: 1, 2, 3, 7, 8, 9, 20, 21, 22, 23, 34, 36) with three different expressions: happy, angry, and neutral (Tottenham et al., 2009). Closed-mouth neutral and open-mouthed happy and angry faces were used as these stimuli were most often correctly identified (see validation data provided by Tottenham and colleagues: http://www.macbrain.org/faq.htm). In order to avoid any influence of race, models were first excluded if they were of African- or Asian-American ethnicity. Six female and six male models whose expressions were identified with the highest accuracy across the three relevant expressions were chosen. This resulted in 36 images of faces. Participants rated each of these faces on one question “How is this person feeling?” on a scale from 1 (extremely angry) to 9 (extremely happy). Each trial began with a 500 ms fixation cross and then the presentation of a face with the question and the rating scale underneath. This remained on screen until participants gave their response. The order in which the faces were presented was randomized.

##### Automatic imitation task

**Figure 1:**
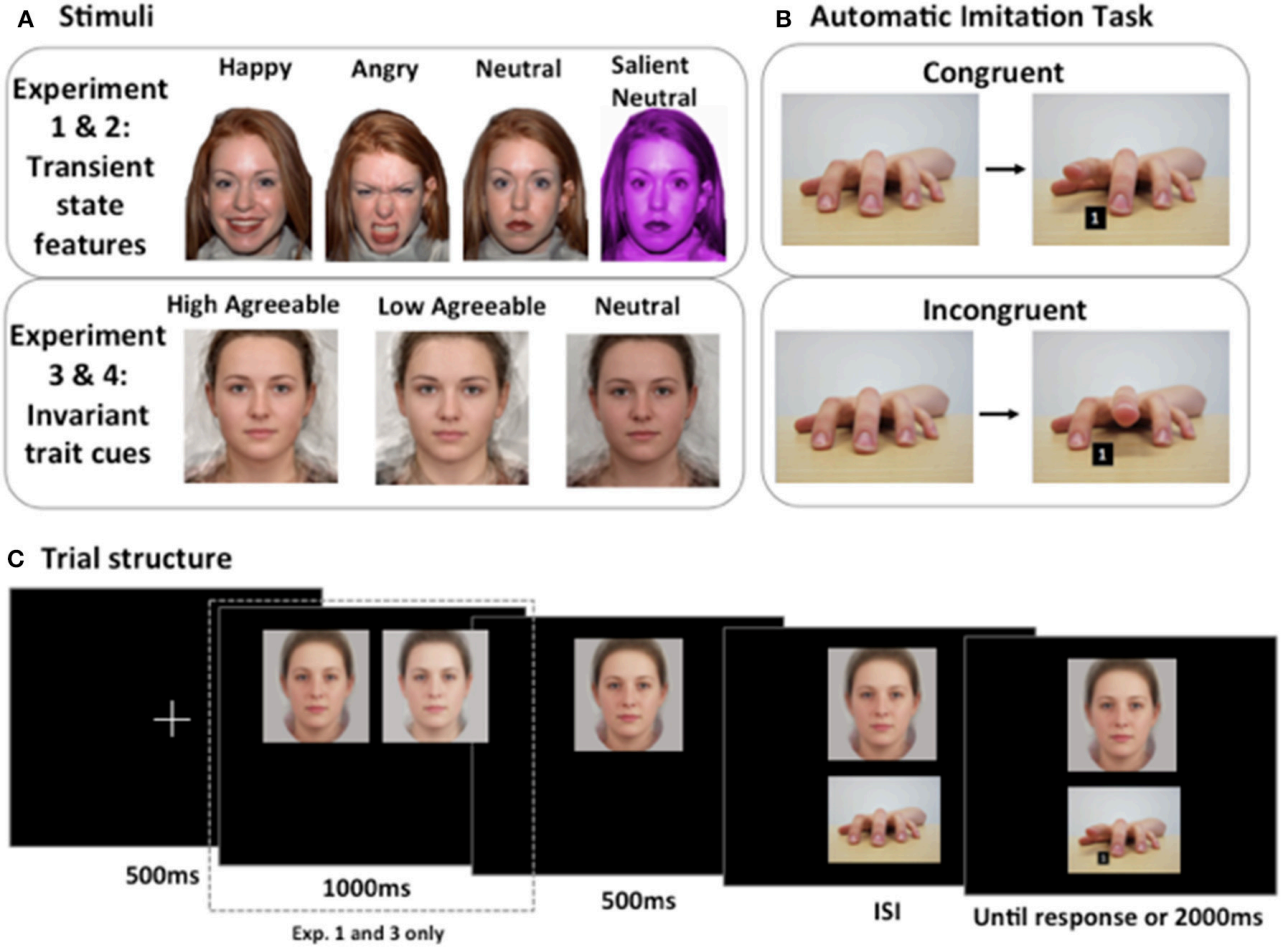
**Stimuli, task, and trial structure for the automatic imitation task. (A)** Stimuli. In Experiments 1 and 2, the hypothesis that transient state cues would influence automatic imitation was tested. In Experiment 1, happy, angry, and neutral individuals were presented. In Experiment 2, an additional condition was added, which was salient but emotionally neutral. In Experiments 3 and 4, the hypothesis that invariant trait cues would influence automatic imitation was tested. In Experiment 3, high and low agreeable composites, as well as neutral composites were presented. In Experiment 4, to focus on the distinction between high and low agreeableness, neutral faces were not presented. **(B)** Automatic imitation task. Congruent trials involved responding to a number cue, whilst simultaneously observing a matching action. Incongruent trials involved responding to a number cue, whilst simultaneously observing a non-matching action. A number one cued participants to lift their index finger, and a number two cued participants to lift their middle finger. **(C)** Trial structure. In Experiments 1 and 3, faces were first paired together, before a single face remained onscreen. Initially pairing faces together in this manner enabled the distinction between facial signals to be highlighted. In Experiments 2 and 4, faces were presented singularly on each trial to avoid possible contamination between different facial signals. ISI, interstimulus interval.

The automatic imitation task was based on the paradigm developed by Brass et al. (2000). Stimuli were a female left hand in a neutral position, resting on a flat surface and four target images of the same hand lifting it's index or middle finger whilst a target number “1” or “2” appeared between the index and middle fingers of the hand. A number “1” cued the participant to lift their index finger and a number “2” cued the participant to lift their middle finger (Figure 1B). Thus, there were four target trial types, two of which were congruent and two of which were incongruent. During congruent trials, the observed hand action was the same as the participants cued action (observed index finger lift and “1,” or observed middle finger lift and “2”) whereas during incongruent trials, the observed hand action and the participants cued action were not the same (observed index finger lift and “2,” or observed middle finger lift and “1”).

Each automatic imitation trial adhered to the following structure (summarized in Figure 1C). Participants were presented with a fixation cross for 500 ms and then a face pair was presented side-by-side for 1000 ms. This presentation of a face pair emphasized the differences between the two faces. Next, one of two faces would disappear and a single face, one of the two shown previously in the face pair, would be shown for 500 ms in the center of the screen. As such, on each expressive trial, a happy and an angry face were initially presented together to highlight differences between them. On neutral trials, two neutral faces were paired together. On expressive trials, for half of the trials a happy face remained onscreen and for half of the trials an angry face remained onscreen. A neutral hand would appear underneath the face for a variable duration (500, 700, or 1000 ms) before the imperative stimulus was presented. The imperative stimulus displayed a finger in a raised position with a number cue between the index and middle finger. The imperative stimulus remained onscreen until participants made their response, but for no longer than 2000 ms. Thus, trial duration varied but was never longer than 5000 ms.

Prior to the start of the task, participants were instructed to hold down the “n” key with the index finger of their right hand and the “m” key with their middle finger. On each trial, upon presentation of a number cue, participants' instructions were to lift their index finger if it was a “1” and their middle finger if it was a “2” as quickly and as accurately as possible. Reaction time (RT) and accuracy rates were recorded. RTs were measured from the presentation of the number cue until participants lifted one of their fingers. Accuracy rates were recorded as the percentage of total trials in which participants made correct responses.

Catch trials were included at the end of 10% of trials. On catch trials, there was an additional 500 ms fixation at the end of the trial followed by the presentation of a single face with the text “Same or different?” underneath. For half of the catch trials, the face was the same as the one that preceded it. This face remained on screen until the participant responded, but for no longer than 10,000 ms. If participants thought the face was the same as the one in the directly preceding trial, they lifted their index finger from the “n” key and if they thought it was different, they lifted their index finger from the “m” key. Accurate performance on catch trials required participants to attend to the face stimuli.

#### Design and procedure

The automatic imitation task employed a 2 × 3 factorial design, with factors of congruency (congruent, incongruent) and face type (angry, happy, neutral). Participants first completed an 8-trial practice of the automatic imitation task, before completing the main automatic imitation task. There were 6 repetitions of each model presented whilst happy, angry, and neutral, thus the main task comprised 216 trials split into 4 equal blocks. Faces were paired so that each happy face was paired with each angry face, resulting in 144 trials that began with a happy-angry pair. Neutral faces were presented in fixed pairs, such that there were two female pairs, two male pairs and two pairs that comprised a male and a female. This resulted in 72 trials that began with a neutral pair. Of the emotionally expressive trials, half were happy and half were angry. For all conditions, congruent and incongruent trials were evenly distributed. For half of the congruent and incongruent trials the face had been presented on the left in the pair previously in the trial. Automatic imitation trials were pseudorandomised such that no face pair was shown consecutively and no target hand image was shown more than three times in succession. Thus, there were 36 trials per condition (happy congruent, happy incongruent, angry congruent, angry incongruent, neutral congruent, neutral incongruent). Once the main task was completed, participants completed the face evaluation task whereby they rated each image on how angry to happy the person appeared to be feeling.

#### Data analysis

The general analysis strategy used throughout these experiments is largely based on the principles of estimation outlined for data analysis in psychological science (Cumming, 2012), which has been endorsed in the (American Psychological Association, 2010) (APA's) *Publication Manual* (APA, 2010). The principles of estimation focus on point and interval estimates as a basis for interpretation wherever possible, rather than primary reliance on null-hypothesis significance testing and *p*-values as the basis for interpretation (Wilkinson, 1999; Kline, 2004). As such, wherever possible inferences are based on effect sizes, confidence intervals (Cumming and Finch, 2005) and meta-analyses (Cumming, 2012). Following suggestions by Cumming (2012), we take a four-step strategy for estimation: (1) Choose one or more effect sizes most relevant to the research question; (2) Place confidence intervals around those effect sizes; (3) Make a figure; (4) Interpret. Finally, wherever possible, we use meta-analyses to combine data from prior relevant studies with our own data in order to provide the most precise estimate of the effects of interest.

In the subsections below, we outline our primary effect sizes of interest. In addition, we estimate each effect size in the following way. For difference scores between two means, we report the effect size and associated 95% CIs in original units (e.g., milliseconds). We also report a standardized effect size (Cohen's d_*z*_) to help compare to other effects in the literature and a Bayes factor (BF_01_) to provide support for the null hypothesis. Estimating effect sizes in this manner is a departure from the traditional statistical approach in psychology, which is based largely on null hypothesis significance testing and the rejection or acceptance of the null hypothesis. By estimating effect sizes and reporting BF_01_, we can offer support for the experimental hypothesis as well as support for the null hypothesis (no difference). To calculate 95% CIs, we use t-distribution values for the confidence co-efficient (Cumming, 2012). Cohen's d_*z*_ is calculated by dividing the mean difference by the standard deviation of the difference (Lakens, 2013). Bayesian analyses are performed in JASP using the paired *t*-test function and reporting BF_01_, which is a ratio of evidence in favor of the null hypothesis over the experimental hypothesis (JASP Team, 2016^2^). Bayes factors provide an estimate of whether the null hypothesis or the experimental hypothesis is more likely given the data. For example, a Bayes factor of BF_01_ = 4 would suggest that the null hypothesis is four times for likely than the experimental hypothesis. To compare to prior studies, we also use ANOVA to calculate partial eta squared (partial η^2^) for effects of interest.

##### Face evaluation

A mean score between 1 and 9, with 1 being extremely angry and 9 being extremely happy, was calculated for each of the three face types (happy, angry, and neutral). We expect differences in ratings between all three means, which we estimate using pair-wise comparisons and one-way ANOVA.

##### Automatic imitation

Prior to analysis, trials were removed if participants gave an incorrect response, lifted their finger from the “n” or the “m” key during the ISI, or took longer than 2000 ms to respond. For each participant, accuracy was calculated as the percentage of correct responses that participants made. For each condition (Happy, Angry, Neutral), we calculated mean average RT and standard error of the mean for congruent and incongruent conditions, as well as the congruency effect (Supplementary Table 1). The congruency effect is calculated by subtracting RT on congruent trials from RT on incongruent trials for each condition.

The key tests of our primary hypothesis are tests for differences in performance on the imitation task, as a function of facial expressions. For all analyses, we calculate difference scores on the congruency effect (CE), and reaction times (RTs) for the congruent and incongruent conditions. As such, differences between angry and happy would be calculated by subtracting the congruency effect for angry from happy ([happy incongruent RT – congruent RT] – [angry incongruent RT – congruent RT]), congruent RT for angry from happy (happy congruent RT – angry congruent RT), and incongruent RT for angry from happy (happy incongruent RT – angry incongruent RT). Analysing congruent and incongruent conditions separately to the CE can help identify the origin of any observed differences in the congruency effect between conditions. The closest prior study (Rauchbauer et al., 2015) showed that happy faces produced a larger CE than angry faces. In addition, happy expressions produced faster congruent RTs than angry expressions and slower incongruent RTs than angry expressions. When these effects were combined into a 2 (face type: angry, happy) × 2 (congruency: congruent, incongruent) repeated measures ANOVA, the effect size for the interaction term (partial η^2^) was 0.33. To compare to this prior work, we first estimate differences between happy and angry expressions using paired differences and by calculating the interaction term of a 2 (face type: angry, happy) × 2 (congruency: congruent, incongruent) repeated measures ANOVA. In addition, as we have included a neutral condition, we also calculate differences from Happy and Angry compared to Neutral, run an ANOVA on CE for the three face types, and calculate the interaction term in a 3 (face type: angry, happy, neutral) × 2 (congruency: congruent, incongruent) repeated measures ANOVA on RT. To interpret these effects, we make a figure that illustrates the difference scores between relevant means and the precision of these estimates using 95% CIs.

In terms of sensitivity to detect effects, a sample of 27 is larger than or equal to the four prior studies investigating how facial signals impact automatic imitation (Table 1). Further, we calculated the power of our different analyses to detect effect sizes similar to that which were observed in the most relevant prior study, which compared the influence of happy and angry faces on the automatic imitation task (Rauchbauer et al., 2015). Rauchbauer and colleagues found the difference in congruency effect between happy and angry expressions had a Cohen's d_z_ of 0.69 (Table 1). In addition, the interaction between congruency and expression in the factorial ANOVA had a partial eta squared (Partial η^2^) of 0.33^3^. We performed two power analyses using G*Power 3.1 (Faul et al., 2009). First, we performed a power analysis for the difference between congruency effects using the *t*-test family of tests and the “Means: difference between two dependent means (matched pairs)” function. The first power analysis showed that using a one-tailed paired *t*-test (e.g., Happy > Angry) with a correlation between conditions of 0.5, we would require 15 participants for 80% power. A further sensitivity test showed that a sample of 27 participants would provide over 95% power to detect a similar size effect to that shown previously. Second, we performed a power analysis for the interaction between congruency and emotion. First, within G*Power, we converted the partial eta squared into an *f*-value (Cohen's *f*), which G*Power requires for power analyses. We then selected the *F*-tests family of tests and the “ANOVA: Repeated measures, within factors” function. The second power analysis showed that a sample of 13 would provide 83% power to detect an interaction of the size observed previously by Rauchbauer et al. (2015). Further, a sample of 27 would provide over 95% power to detect a similar effect to that previously found (Partial η^2^ of 0.33).

### Results

#### Face evaluation

**Figure 2:**
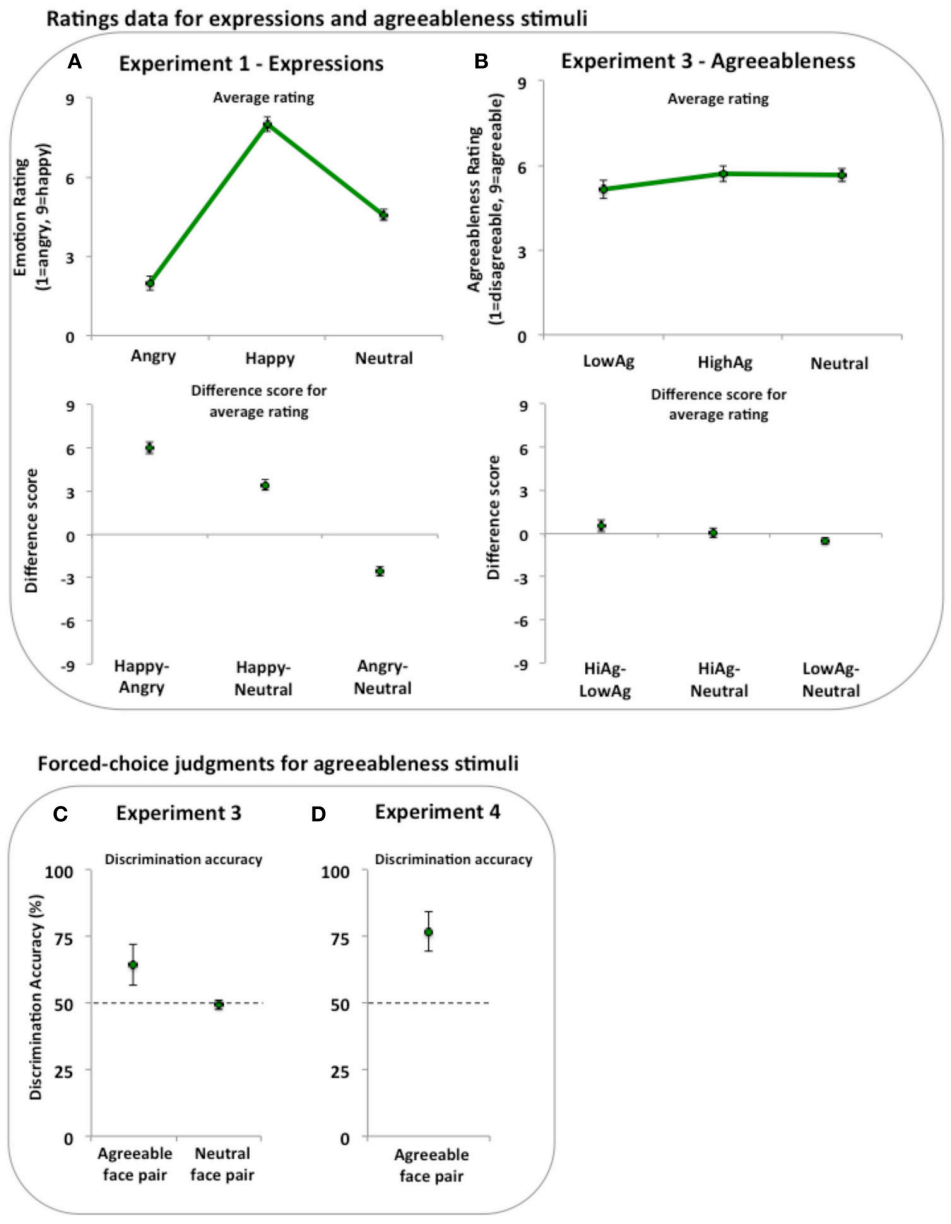
**Ratings and judgments of stimuli across Experiments 1, 3, and 4. (A)** In a ratings task, happy faces were rated happier than angry and neutral faces. In addition, the angry faces were rated as angrier than the neutral faces. **(B)** In a ratings task, low agreeable faces were rated as less agreeable than the high agreeable and neutral faces. High agreeable and neutral faces were rated similarly. **(C)** In a 2 alternative-forced-choice (2AFC) task, discrimination performance was greater than chance (dashed line) when judging between high and low agreeable face pairs, but not when judging between neutral face pairs. **(D)** In a 2AFC task, discrimination performance was greater than chance (dashed line) when judging between high and low agreeable face pairs. Error bars represent 95% confidence intervals (95% CIs).

Means and difference scores with 95% CIs are displayed in Figure 2A. Based on a 9-point scale ranging from angry to happy, the difference score was 6.00 (CI = [5.59, 6.42]) with effect size d_z_ = 5.53 between Happy and Angry, 3.43 (CI = [3.05, 3.81]) with effect size d_z_ = 3.53 between Happy and Neutral and −2.58 (CI = [−2.92, −2.24]) with effect size d_z_ = −2.95 between Angry and Neutral. These data show that ratings of facial expressions differ in the direction expected.

#### Automatic imitation

Trials were removed if participants gave an incorrect response (2.97%), lifted their finger from the “n” or the “m” key during the ISI (0.02%), or took longer than 2000 ms to respond (0.14%). Accuracy on catch trials was 83.67% (CI = [79.67, 87.67]; Cohen's d_z_ = 3.12), with chance performance at 50%.

**Figure 3:**
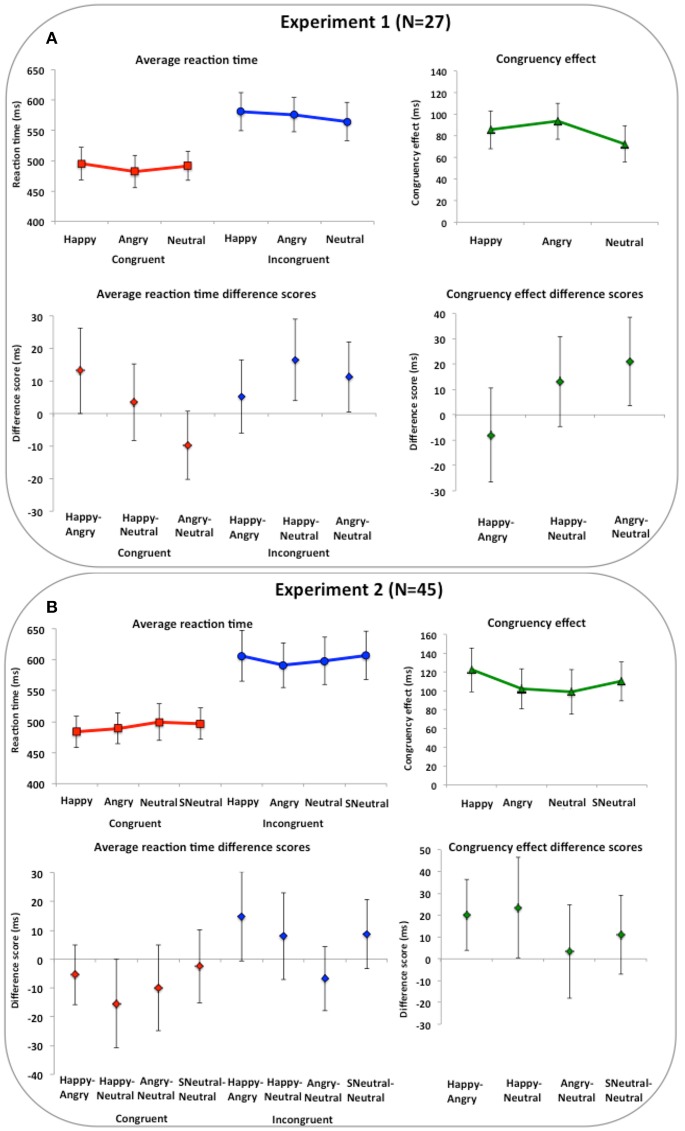
**Imitation task results for Experiments 1 and 2 (expressions)**. Imitation task results for Experiments 1 and 2 (expressions). In upper panels, reaction times and congruency effects (incongruent minus congruent) per condition are shown. In lower panels, difference scores between conditions for congruent, incongruent and congruency effects are shown. **(A)** Results from Experiment 1 show a similar influence of happy and angry faces compared to neutral on imitation with both leading to greater interference on incongruent trials. **(B)** Results from Experiment 2 show happy expressions are related to greater imitation than angry or neutral expressions and that this is unlikely to be a general-purpose mechanism as the salient neutral face was not related to greater imitation than the neutral face. Abbreviations: SNeutral, Salient Neutral. Error bars are 95% confidence intervals.

Mean average RT and standard error of the mean for congruent and incongruent conditions, as well as the CE are reported in Supplementary Table 1. In Table 2 and Figure 3A, estimation information on key contrasts are reported and illustrated. Our key contrasts involve comparing performance on the imitation tasks across different expressions. First, we estimate differences between happy and angry expressions, which closely follows prior work (Rauchbauer et al., 2015). Differences between happy compared to angry expressions were calculated for the CE −7.96 (CI = [−26.50, 10.58]) with effect size d_z_ = −0.17 and Bayes Factor BF_01_ = 3.44, the congruent condition 13.16 (CI = [−0.01, 26.33]) with effect size d_z_ = 0.40 and Bayes Factor BF_01_ = 0.80 and the incongruent condition 5.20 (CI = [−6.11, 16.52]) with effect size d_z_ = 0.18 and Bayes Factor BF_01_ = 3.27. We also entered the RT data into a 2 (face type: angry, happy) × 2 (congruency: congruent, incongruent) ANOVA, and report the face type by congruency interaction [*F*_(1, 26)_ = 0.78, *p* = 0.385, $\eta_{p}^{2}$ = 0.029].

**Table 2:** **Results from Experiments 1 and 2 (facial expressions)**.

**Study/Sample size/Manipulation**	**Contrast**	**Mean difference (ms)**		**95% confidence intervals**	**Cohen's d_z_**	**Bayes factor BF_01_**
Experiment 1	Happy > Angry					
*N* = 27		CE	−7.96	[−26.50, 10.58]	−0.17	3.44
Facial expressions		Congruent	13.16	[−0.01, 26.33]	0.40	0.80
		Incongruent	5.20	[−6.11, 16.52]	0.18	3.27
	Happy > Neutral					
		CE	13.05	[−4.73, 30.84]	0.29	1.79
		Congruent	3.43	[−8.34, 15.21]	0.12	4.16
		Incongruent	16.49	[3.90, 29.07]	0.52	0.25
	Angry > Neutral					
		CE	21.01	[3.62, 38.41]	0.48	0.38
		Congruent	−9.73	[−20.23, 0.77]	−0.37	1.02
		Incongruent	11.29	[0.45, 22.12]	0.41	0.70
Experiment 2	Happy > Angry					
*N* = 45		CE	20.16	[3.87, 36.46]	0.37	0.39
		Congruent	−5.45	[−15.82, 4.92]	−0.16	3.67
Facial expressions		Incongruent	14.72	[−0.73, 30.17]	0.29	1.16
	Happy > Neutral					
		CE	23.39	[0.39, 46.40]	0.31	0.92
		Congruent	−15.43	[−30.75, −0.11]	−0.30	0.96
		Incongruent	7.96	[−7.15, 23.08]	0.16	3.65
	Angry > Neutral					
		CE	3.23	[−18.12, 24.58]	0.05	5.92
		Congruent	−9.98	[−24.82, 4.85]	−0.20	2.64
		Incongruent	−6.75	[−17.89, 4.39]	−0.18	3.09
	Salient Neutral > Neutral					
		CE	11.10	[−7.02, 29.21]	0.18	3.04
		Congruent	−2.44	[−15.15, 10.28]	−0.06	5.77
		Incongruent	8.66	[−3.23, 20.65]	0.22	2.32

Second, we compared emotional expressions to neutral, separately for happy and angry expressions. Differences between happy compared to neutral expressions were calculated for the CE 13.05 (CI = [−4.73, 30.84]) with effect size d_z_ = 0.29 and Bayes Factor BF_01_ = 1.79, the congruent condition 3.43 (CI = [−8.34, 15.21]) with effect size d_z_ = 0.12 and Bayes Factor BF_01_ = 4.16 and the incongruent condition 16.49 (CI = [3.90, 29.07]) with effect size d_z_ = 0.52 and Bayes Factor BF_01_ = 0.25. Differences between angry compared to neutral expressions were calculated for the CE 21.01 (CI = [3.62, 38.41]) with effect size d_z_ = 0.48 and Bayes Factor BF_01_ = 0.38, the congruent condition −9.73 (CI = [−20.23, 0.77]) with effect size d_z_ = −0.37 and Bayes Factor BF_01_ = 1.02 and the incongruent conditions 11.29 (CI = [0.45, 22.12]) with effect size d_z_ = 0.41 and Bayes Factor BF_01_ = 0.70. We also entered the RT data into a 3 (face type: angry, happy, neutral) × 2 (congruency: congruent, incongruent) ANOVA, and report the face type by congruency interaction [*F*_(2, 52)_ = 2.97, *p* = 0.060, $\eta_{p}^{2}$ = 0.102]. Separate ANOVAs were subsequently performed on congruent trials [*F*_(2, 52)_ = 2.80, *p* = 0.070, $\eta_{p}^{2}$ = 0.097] and incongruent trials [*F*_(2, 52)_ = 4.46, *p* = 0.016, $\eta_{p}^{2}$ = 0.147].

### Discussion

This is the first experiment to compare the influence of positive, negative, and neutral facial signals on a measure of automatic imitation. We find that happy and angry expressions produced greater interference to the incongruent condition than neutral expressions. Following benchmark interpretations of Cohen's d, the effect sizes are small-to-medium or medium in size (Cohen's d_z_ = 0.41, 0.52). Considered in isolation, these data are consistent with view that imitation may work to increase affiliation with a happy person (Chartrand and Lakin, 2013), but also to appease someone who appears to be angry (Rauchbauer et al., 2016).

When we directly compare happy to angry expressions, we do not provide support for the closest previous study (Rauchbauer et al., 2015). Rauchbauer et al. (2015) showed differences between happy and angry expressions on the CE, congruent and incongruent conditions. In the present experiment, 95% CIs for the difference between happy and angry overlap with zero for the CE and the incongruent condition and show small effect sizes, whereas the difference on the congruent condition is in the opposite direction to data previously reported by Rauchbauer et al. (2015). Thus, our initial estimate of the impact of happy compared to angry expressions is discrepant with the closest prior estimate. However, Experiment 1 was not intended to be a direct replication of Rauchbauer and colleagues work, but features of the design are similar enough for relatively straightforward comparisons to be made. There are still some key differences, however. In order to highlight the differences between expressions, the design of Experiment 1 did not clearly separate happy and angry expressions. On expressive trials, participants saw both happy and angry expressions within seconds of performing the imitation task, which makes it difficult to separate the influence of positive and negative facial signals on imitation. To separate the influence of happy and angry expressions, only one face will be presented prior to the imitation task in Experiment 2. This design also more closely replicates the approach taken by Rauchbauer et al. (2015).

In addition, the results from Experiment 1 do not demonstrate if the influence of expression on imitation is tied to emotional state signals from faces *per se*, or if it indexes a more general-purpose mechanism. For instance, according to a general-purpose mechanism, expressive signals may capture attentional resources more than neutral facial signals, which could contribute to stronger interference in the incongruent condition. To further test the specificity of this effect, Experiment 2 includes an additional neutral condition. Finally, the effect sizes observed in Experiment 1 are smaller than previously reported by Rauchbauer et al. (2015) and we therefore wanted to increase sensitivity of our main test by increasing the sample size to make it as close to 50 as possible.

## Experiment 2

### Introduction

Experiment 2 separates the influence of happy and angry faces by presenting faces singularly on every trial. By doing so, it will more directly replicate the design employed by Rauchbauer et al. (2015). Additionally, in Experiment 2 an extra neutral condition will be added, which has a salient facial feature (non-biological skin color), but remains emotionally neutral (Figure 1A). The inclusion of an additional condition will help distinguish whether the influence of expressive faces is due to the social signals they convey or whether the influence is indicative of a more general-purpose mechanism. If the influence of expression is due to social signals from the face *per se*, it is expected that expressive faces will elicit a greater imitation behavior than the new salient neutral condition. By contrast, if the influence of facial expression operates through a more general-purpose mechanism, one that is not specifically tied to emotional states, then RT patterns following expressive faces should be similar to the new neutral condition.

### Methods

#### Participants

Forty-nine Bangor University students participated for course credit. Four participants were removed from the sample as their accuracy on congruent (*n* = 2) or incongruent (*n* = 2) trials was >3SD from the group mean. Of the remaining 45 (34 female, 11 male; *M*_age_ = 19.33 years, *SD* = 2.09) all had normal or corrected-to-normal vision and 43 were right handed as measured by the Edinburgh Handedness Inventory. This measure showed that two participants were ambidextrous, although they were included in the sample as they reported being predominantly right-hand users. The study complied with the guidelines set by the Research Ethics and Governance Committee of the School of Psychology at Bangor University.

#### Task and stimuli

Stimuli were the same images used in Experiment 1 with the addition of a second category of neutral images. The additional neutral stimuli comprised the 12 neutral face images modified so that they were more salient (non-biological skin color), but remained emotionally neutral. Thus, there were a total of 48 face images, four variations of 12 models that were angry, happy, neutral, and salient neutral.

The task and trial structure was the same as in Experiment 1 without presentation of face pairs (see Figure 1C). Instead, faces were presented singularly during each trial. There were 192 total trials and 8 trial types (congruent and incongruent, for each of the four face types), thus there were 24 trials per trial type. Randomization and ISI were the same as in Experiments 1.

#### Design and procedure

The automatic imitation task employed a 2 × 4 factorial design, with factors of congruency (congruent, incongruent) and face type (happy, angry, neutral, salient neutral). Participants first completed an 8-trial practice of the automatic imitation task, before completing the main automatic imitation task following the same procedure as Experiment 1.

#### Data analysis

Data were processed and analyzed in the same way as Experiment 1. Two sensitivity analyses were performed in G*Power to show the extent to which a sample size of 45 would increase sensitivity to detect our primary effects of interest. Using a one-tailed paired *t*-test (e.g., Happy > Angry) with a correlation between conditions of 0.5, we would have 80% power to detect a Cohen's d of 0.38. In addition, a sample of 45 would provide 80% power to detect an interaction between face-type and congruency of partial η^2^ = 0.08.

### Results

#### Automatic imitation

As in Experiments 1, prior to analysis, trials were removed if participants gave an incorrect response (4.53%), lifted their finger from the “n” or the “m” key during the ISI (0.06%), or took longer than 2000 ms to respond (0.24%). Accuracy on catch trials was 84.56% (CI = [80.85, 88.26]; Cohen's d_*z*_ = 2.72), with chance performance being 50%.

Mean average RT and standard error of the mean for congruent and incongruent conditions, as well as the CE are reported in Supplementary Table 1. In Table 2 and Figure 3B, estimation information on key contrasts are reported and illustrated. Our key contrasts involve comparing performance on the imitation tasks across different expressions. First, we estimate differences between happy and angry expressions, which closely follows prior work (Rauchbauer et al., 2015). Differences between happy compared to angry expressions were calculated for the CE 20.16 (CI = [3.87, 36.46]) with effect size d_z_ = 0.37 and Bayes Factor BF_01_ = 0.39, the congruent condition −5.45 (CI = [−15.82, 4.92]) with effect size d_z_ = −0.16 and Bayes Factor BF_01_ = 3.67 and the incongruent condition 14.72 (CI = [−0.73, 30.17]) with effect size d_z_ = 0.29 and Bayes Factor BF_01_ = 1.16. We also entered the RT data into a 2 (face-type) × 2 (congruency) repeated measures ANOVA, and report the face type by congruency interaction [*F*_(1, 44)_ = 6.22, *p* = 0.016 $\eta_{p}^{2}$ = 0.124] for direct comparison to prior work (Rauchbauer et al., 2015).

Second, we compared happy, angry and salient neutral conditions to neutral. Differences between happy compared to neutral expressions were calculated for the CE 23.39 (CI = [0.39, 46.40]) with effect size d_z_ = 0.31 and Bayes Factor BF_01_ = 0.92, the congruent condition −15.43 (CI = [−30.75, −0.11]) with effect size d_z_ = −0.30 and Bayes Factor BF_01_ = 0.96 and the incongruent condition 7.96 (CI = [−7.15, 23.08]) with effect size d_z_ = 0.16 and Bayes Factor BF_01_ = 3.65. Differences between angry compared to neutral expressions were calculated for the CE 3.23 (CI = [−18.12, 24.58] d_z_ = 0.05 and Bayes Factor BF_01_ = 5.92, the congruent condition −9.98 (CI = [−24.82, 4.85]) with effect size d_z_ = −0.20 and Bayes Factor BF_01_ = 2.64 and the incongruent conditions −6.75 (CI = [−17.89, 4.39]) with effect size d_z_ = −0.18 and Bayes Factor BF_01_ = 3.09. Differences between salient neutral compared to neutral expressions were calculated for the CE 11.10 (CI = [−7.02, 29.21]) with effect size d_z_ = 0.18 and Bayes Factor BF_01_ = 3.04, the congruent condition −2.44 (CI = [−15.15, 10.28]) with effect size d_z_ = −0.06 and Bayes Factor BF_01_ = 5.77 and the incongruent conditions −8.66 (CI = [−3.23, 20.65]) with effect size d_z_ = 0.22 and Bayes Factor BF_01_ = 2.32. We also entered the RT data into a 4 (face type: angry, happy, neutral, salient neutral) × 2 (congruency: congruent, incongruent) ANOVA, and report the face type by congruency interaction [F_(3, 132)_ = 2.55, *p* = 0.058, $\eta_{p}^{2}$ = 0.055]. Separate ANOVAs were subsequently performed on congruent trials [*F*_(3, 132)_ = −2.7, *p* = 0.048, $\eta_{p}^{2}$ = 0.058] and incongruent trials [*F*_(3, 132)_ = 2.25, *p* = 0.086, $\eta_{p}^{2}$ = 0.049].

#### Meta-analysis of expressions data

To provide the most precise estimate of our effects of interest we performed a meta-analysis using Exploratory Software for Confidence Intervals (ESCI; Cumming, 2012). ESCI is free software, which runs in Microsoft Excel and allows data for several studies to be meta-analyzed. Each study included in a meta-analysis makes a weighted contribution to the grand estimate of all studies. The weighting of each study's data is calculated as a function of sample size and variability of the estimate. Therefore, studies with larger samples and smaller variability have a higher weighting than studies with smaller samples and larger variability.

We performed meta-analyses for three separate effects (Happy > Angry, Happy > Neutral, Angry > Neutral). For all effects, we included estimates of the congruency effect, as well as the congruent and incongruent conditions, and for each effect of interest we included data from all available studies to date^4^. For the Happy > Angry, we included data from six experiments including Rauchbauer et al. (1 experiment: 2015; 3 experiments: 2016), as well as Experiments 1 and 2 from the current study. Happy and angry conditions were collapsed across the other conditions in previous experiments (i.e., group membership or threat level). For the Happy > Neutral, we included data from Experiments 1 and 2 from the current study. For the Angry > Neutral, we included data from three experiments including Crescentini et al. (2011), as well as Experiments 1 and 2 from the current study. As recommended by Cumming (2012), in all cases we used a random effects model to estimate the mean estimate across studies.

**Figure 4:**
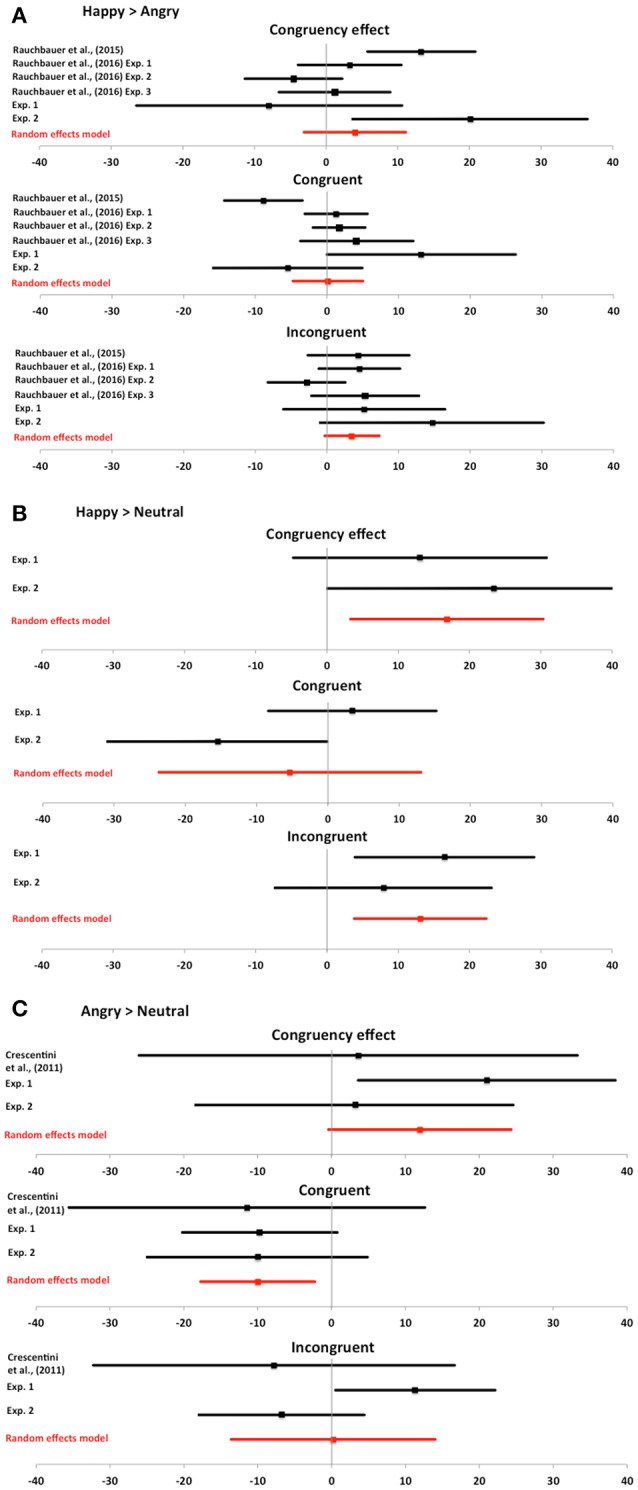
**Meta-analyses for expression data**. Meta-analyses for expression data. Bars represent point estimates and 95% confidence intervals for the effect of interest from each study in the meta-analysis, as well as the combined random effects model. Whilst this manuscript was under review an additional paper was published (Rauchbauer et al., 2016). The authors kindly provided their data to use in our meta-analysis for completeness of reporting previous effects. For each effect of interest, we report meta-analytical data on the congruency effect, as well as congruent and incongruent conditions. **(A)** Happy minus angry. Meta-analyses show that happy expressions are most consistently related to greater imitation than angry expressions, driven primarily by differences in incongruent RTs. **(B)** Happy minus neutral. Meta-analysis shows that happy expressions are related to greater imitation than neutral expressions, again driven primarily by differences in incongruent RTs. **(C)** Angry minus neutral. Meta-analysis shows that angry expressions are related to greater imitation than neutral expressions, driven primarily by faster responses on congruent trials.

We illustrate the results of the meta-analysis in Figure 4 using forest plots. If we focus on results from the random effects model (shown in red), which represents our best estimate to date, several patterns of data emerge. Happy expressions have a common impact on automatic imitation compared to angry and neutral conditions. In both cases, there is a positive difference in congruency effect, which is primarily driven by differences on the incongruent condition (Figures 4A,B). Angry expressions compared to neutral also lead to a positive difference in congruency effect, but this is largely driven by faster responses on congruent conditions (Figure 4C).

### Discussion

In Experiment 2 when prosocial and antisocial expressions are clearly separated, we show that happy expressions lead to greater interference to task performance than angry and neutral expressions. In addition, the impact of a happy expression on imitation seems unlikely to be a general-purpose influence. A salient but emotionally neutral face produced no clear impact on automatic imitation. We must be cautious however, as saliency was not included as a full factor in our design and thus future work could more rigorously test whether the impact of emotional facial expression on imitation when compared against a neutral facial expression could be explained by increased salience of the emotionally expressive faces, or whether the modulation is driven by the social content of emotional facial expressions.

When data for Experiment 2 are compared to Rauchbauer et al. (2015), more similarities emerge between the two datasets than in Experiment 1. In terms of the congruency effect, the effects are similar, although smaller in magnitude. Indeed, the standardized effect size is nearly half the size of that reported previously (Cohen's d_z_ 0.37 vs. 0.69). A further similarity is the effect on the incongruent condition, which was similar in size and direction to the prior report (Cohen's d_z_ 0.29 vs. 0.25). However, in the current study there was only a small effect on the congruent condition and the 95% CIs overlapped with zero, whereas previously it was a much larger effect (Cohen's d_z_ −0.16 vs. −0.65). Further, the face-type by congruency interaction is a third of the size reported by Rauchbauer and colleagues ($\eta_{p}^{2}$ 0.12 vs. 0.33). In sum, we provide further support for the proposal that happy expressions influence imitation more than angry, but we show that the size of the influence is smaller than initially reported and that the effect is less consistent on congruent than incongruent conditions.

The meta-analytical data (Figure 4) show distinct influences of happy and angry facial expressions on imitation. Across all studies to date, the best estimate of the influence of angry expressions on imitation is that imitation is increased as a result of faster responses when an observed action matches the participant's cued action (congruent trials). By contrast, imitation increased with perception of happy expressions compared to angry or neutral expressions as a result of increased interference on incongruent trials. Therefore, these meta-analytical data suggest that expressions may influence automatic imitation via different mechanisms. Angry expressions facilitate the congruent condition, whereas happy expressions lead to greater interference on the incongruent condition. To date, however, the evidence is limited to a few studies and more data points are required for a more robust understanding of the influence of expression on imitation. To aid further meta-analyses and build a more comprehensive picture, future studies should report differences on congruent and incongruent conditions as well as the congruency effect.

Including the current study, four studies to date have investigated the impact of expressions on imitation, but no study to our knowledge has investigated how invariant features of the face, which signal trait-levels of agreeableness influence imitation. Experiments 3 and 4 take a similar approach to the first two experiments in terms of experimental design, but use different facial cues.

## Experiment 3

### Methods

#### Participants

Thirty-one students (24 female, 7 male; *M*_age_ = 20.1 years, *SD* = 3.36) from Bangor University participated for course credit. All participants had normal or corrected-to-normal vision and reported being right handed. The Edinburgh Handedness Inventory showed that two were ambidextrous. However, they were retained in the sample, as they were predominantly right-hand users. No participants were removed. All participants gave written, informed consent and were fully debriefed. The Research Ethics and Governance Committee of the School of Psychology at Bangor University granted ethical approval.

#### Task and stimuli

##### Face evaluation tasks

Over several years of data collection at Bangor University, participants have been photographed whilst holding an emotionally neutral expression before completing self-report measures of various personality and subclinical traits (Kramer and Ward, 2010; Jones et al., 2012; Scott et al., 2013). We used these existing datasets to generate average images of individuals that are indicative of high levels of agreeableness and low levels of agreeableness. Composite face images were made that represent high and low agreeableness by using a software package that enables multiple individual faces to be combined into one average face (JPyschomorph; Tiddeman et al., 2001). Face images from 15 individuals who reported the highest levels of agreeableness were morphed into one average high agreeable composite. The same morphing procedure was also performed on the 15 individuals who reported the lowest levels of agreeableness. Previous reports demonstrate a correct consensus for these composites across a range of inventory questions for trait agreeableness (Kramer and Ward, 2010).

Composite face images were created from one of three different datasets. Pair one was taken from Kramer and Ward (2010), pair two was taken from the faces photographed for Jones et al. (2012) and pair three was from the faces photographed for Scott et al. (2013). This resulted in three pairs of high-low agreeable composite images with each pair including one high and one low agreeable face. To produce neutral images, a high agreeable composite and a low agreeable composite image were averaged together. Four neutral images were created and paired together into two neutral pairs. In total, therefore, ten individual composite images of faces were used: three were high agreeable, three were low agreeable and 4 were neutral in terms of agreeableness features (Figure 1A).

Participants completed two face evaluation tasks to examine whether they could accurately judge agreeableness from these faces. The first face evaluation task was a 20-trial two-alternative forced choice (2AFC) task. Participants' viewed five face pairs, three of which comprised a high and a low agreeable face and two pairs comprised neutral faces. On each trial, participants were presented with a face pair and the task was to choose which face best represented one of the four agreeableness-relevant statements from the mini International Personality Item Pool (mini-IPIP; Donnellan et al., 2006). At the beginning of the 2AFC section, participants saw a fixation cross for 1500 ms, followed by presentation of a pair of faces with a question underneath. The face pair and question remained on screen until the participants responded. Therefore, responses were not speeded, however, participants were encouraged to respond with their initial reaction, or “gut instinct,” to the stimuli. Participants were asked to indicate which of the faces best matched the statement underneath by pressing the “n” key for the left face and the “m” key for the right face.

On each trial, the high and the low agreeable face was presented next to each other on the screen with the high agreeable face randomly on the left or the right of the pair. It was expected that for pairs comprising a high and a low agreeable face, participants would accurately discriminate them at a level significantly greater than chance (Penton-Voak et al., 2006; Todorov et al., 2008, 2015; Kramer and Ward, 2010). For the neutral face pairs, which were not trait-diagnostic, performance should be at chance level.

The second face evaluation task was a 40-trial ratings task where participants rated all 10 faces on the four agreeableness-relevant statements from the mini-IPIP in a random order. In this task, each face was presented with a statement underneath. The statement remained on screen until participants had given a response. Participants' task was to rate, based on “gut instinct,” how well they agreed that the face matched the statement using the number keys 1-9, where 1 was strongly disagree and 9 was strongly agree.

##### Automatic imitation task

In order to emphasize the distinction between faces, the task and trial structure was the same as Experiment 1 (Figure 1C). As such, on each trait trial, a high and a low agreeable face were initially presented together to highlight differences between them. On neutral trials, two neutral faces were paired together. After this paired presentation phase, one face would disappear leaving a single face presented centrally. On traits trials, half of the trials a high agreeable face remained onscreen and half of the trials a low agreeable face remaining onscreen.

#### Design and procedure

The automatic imitation task employed a mixed 2 × 3 factorial design, with factors of congruency (congruent, incongruent) and face type (high agreeable, low agreeable, neutral). There were 16 repetitions of each face, resulting in 96 trait representative and 64 trait neutral trials. The design and procedures were the same as in Experiment 1, except for the number of trials, which changed due to the lower number of face images.

Participants completed an 8-trial practice of the automatic imitation task, before completing three tasks. Before the imitation task proper, participants completed the face ratings task and a 20-trial 2AFC task. Participants completed four 40-trial blocks of the automatic imitation task. Blocks of the automatic imitation task alternated with another three blocks of the 2AFC task. The function of alternating between the tasks was to highlight to participants the trait diagnostic value of the faces throughout the experiment. After the last automatic imitation block, participants completed a final 2AFC and a second face ratings task.

#### Data analysis

##### Face evaluation

The percentage of correct responses from the trials where participants viewed pairs of high and low agreeable faces was calculated. This was compared to the percentage that participants would be expected to get if they were performing at chance (i.e., at 50% as it was a 2AFC task). For pairs of neutral faces, the percentage of trials that participants chose one face from each neutral pair consistently as the high agreeable face was calculated. The percentage for trait representative trials and trait neutral trials was compared to chance (i.e., 50%).

For the rating task, the mean score was calculated for the extent to which participants agreed that each face type (high, low, and neutral) looked agreeable on the scale from 1 to 9. We expect differences in ratings between all three means, which we estimate using pair-wise comparisons and one-way ANOVA.

##### Automatic imitation

Data were processed in the same way as in the previous experiments. A sensitivity analysis performed in G*Power showed that using a one-tailed paired *t*-test (e.g., High > Low Agreeable) with a correlation between conditions of 0.5, a sample of 31 we would have 80% power to detect a Cohen's d of 0.46. Further, a sample of 31 would provide over 80% power to detect an interaction between face-type and congruency of partial η^2^ = 0.15.

Firstly, this analysis was carried out for all participants. Then, the same analysis was performed on a subset of participants who showed high accuracy in the 2AFC face evaluation task. This analysis would assess whether trait detection accuracy influences imitation.

### Results

#### Face evaluation

Results from the face discrimination task (Figure 2C) showed that participants were more accurate than chance (64.46% correct; CI = [56.91, 72.02]; Cohen's d_z_ = 0.67) when discriminating high and low agreeable pairs. When discriminating neutral pairs, participants' chose the neutral faces at a rate no different than chance (49.44% correct; CIs [47.71, 51.16]; Cohen's d_z_ = −0.11).

Means and difference scores with 95% CIs are displayed in Figure 2B. Data were collapsed across the two ratings blocks and the differences between ratings of each face type were estimated. The one-way ANOVA showed a difference in ratings between the face types [*F*_(2, 60)_ = 6.47, *p* = 0.003, $\eta_{p}^{2}$ = 0.177]. Based on a 9-point scale ranging from Low to High Agreeable, the difference score was 0.55 (CI = [0.12, 0.97]) with effect size d_z_ = 0.45 between High and Low Agreeable, 0.04 (CI = [−0.26, 0.35]) with effect size d_z_ = 0.05 between High Agreeable and Neutral, and −0.51 (CI = [−0.74, −0.27]) with effect size d_z_ = −0.75 between Low Agreeable and Neutral. These data show that ratings of agreeableness differ in the direction expected between Low Agreeable and both High Agreeable and Neutral faces.

#### Automatic imitation

Prior to analysis, trials were removed if participants gave an incorrect response (5%), lifted their finger from the “n” or the “m” key during the ISI (0.06%), or took longer than 2000 ms to respond (0.18%). Accuracy on catch trials was 61.90% (CI = [57.90, 65.89]; Cohen's d_z_ = 1.05) with chance performance at 50%.

**Figure 5:**
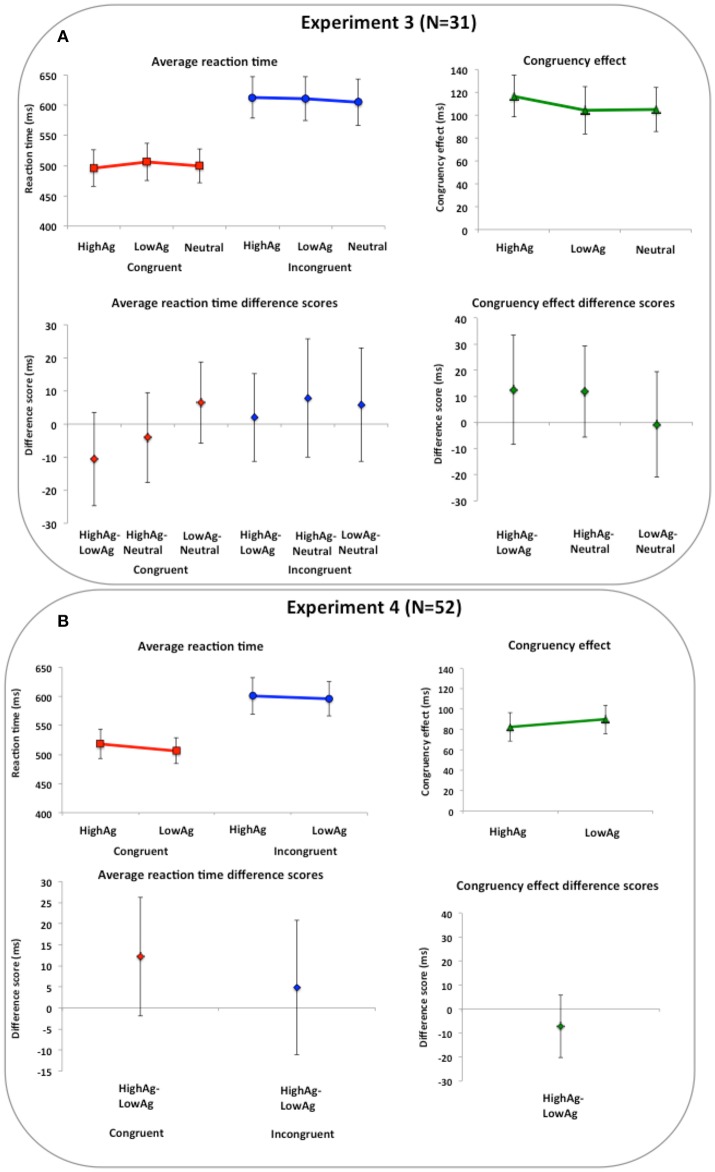
**Imitation task results for Experiments 3 and 4 (agreeableness)**. Imitation task results for Experiments 3 and 4 (trait agreeableness). In upper panels, reaction times and congruency effects (incongruent minus congruent) per condition are shown. In lower panels, difference scores between conditions for congruent, incongruent and congruency effects are shown. **(A)** Results from Experiment 3 show that perception of high and low agreeable trait signals did not influence imitation performance compared to neutral face signals. **(B)** Results from Experiment 4 show that even when high and low agreeable trait signals were clearly separated there was no difference in their impact on imitation performance. Error bars are 95% confidence intervals.

Mean average RT and standard error of the mean for congruent and incongruent conditions, as well as the CE are reported in Supplementary Table 1. In Table 3 and Figure 5A, estimation information on key contrasts are reported and illustrated. Our key contrasts involve comparing performance on the imitation tasks across different face-types. Differences between high agreeable compared to low agreeable faces were calculated for the CE 12.63 (CI = [−8.28, 33.53]) with effect size d_z_ = 0.22 and Bayes Factor BF_01_ = 2.62, the congruent condition −10.60 (CI = [−24.68, 3.47]) with effect size d_z_ = −0.27 and Bayes Factor BF_01_ = 1.81 and the incongruent condition 2.02 (CI = [−11.29, 15.34]) with effect size d_z_ = 0.06 and Bayes Factor BF_01_ = 5.05. We also entered the RT data into a 2 × 2 ANOVA, and report the face type by congruency interaction [*F*_(1, 30)_ = 1.52, *p* = 0.023, $\eta_{p}^{2}$ = 0.048].

**Table 3:** **Results from Experiments 3 and 4 (invariant facial features)**.

**Study/Sample size/Manipulation**	**Contrast**	**Mean difference (ms)**		**95% confidence intervals**	**Cohen's d_z_**	**Bayes factor BF_01_**
Experiment 3	High > Low Agreeable					
*N* = 31		CE	12.63	[−8.28, 33.53]	0.22	2.62
Invariant facial features (Traits) High,		Congruent	−10.60	[−24.68, 3.47]	−0.27	1.81
low, and neutral agreeable		Incongruent	2.02	[−11.29, 15.34]	0.06	5.00
	High > Neutral					
		CE	11.96	[−5.54, 29.45]	0.25	2.12
		Congruent	−4.12	[−17.66, 9.42]	−0.11	4.37
		Incongruent	7.84	[−9.99, 25.67]	0.16	3.61
	Low > Neutral					
		CE	−0.67	[−20.84, 19.50]	−0.01	5.21
		Congruent	6.48	[−5.82, 18.78]	0.19	3.08
		Incongruent	5.81	[−11.39, 23.02]	0.12	4.19
Experiment 4	High > Low Agreeable					
*N* = 52		CE	−7.30	[−20.29, 5.70]	−0.15	3.64
		Congruent	12.14	[−1.98, 26.26]	0.24	1.66
Invariant facial features		Incongruent	4.84	[−11.15, 20.84]	0.08	5.55

Second, we compared trait representative to neutral faces, separately for high and low agreeable faces. Differences between high agreeable compared to neutral were calculated for the CE 11.96 (CI = [−5.54, 29.45]) with effect size d_z_ = 0.25 and Bayes Factor BF_01_ = 2.12, the congruent condition −4.12 (CI = [−17.66, 9.42]) with effect size d_z_ = −0.11 BF_01_ = 4.37 and the incongruent condition 7.84 (CI = [−9.99, 25.67]) with effect size d_z_ = 0.16 and Bayes Factor BF_01_ = 3.61. Differences between low agreeable compared to neutral faces were calculated for the CE −0.67 (CI = [−20.84, 19.50]) with effect size d_z_ = −0.01 and Bayes Factor BF_01_ = 5.21, the congruent condition 6.48 (CI = [−5.82, 18.78]) with effect size d_z_ = −0.19 and Bayes Factor BF_01_ = 3.08 and the incongruent condition 5.81 (CI = [−11.39, 23.02]) with effect size d_z_ = 0.12 and Bayes Factor BF_01_ = 4.19. We also entered the RT data into a 3 (face type: high agreeable, low agreeable, neutral) × 2 (congruency: congruent, incongruent) ANOVA, and report the face type by congruency interaction [*F*_(2, 60)_ = 1.10, *p* = 0.340, $\eta_{p}^{2}$ = 0.035]. Separate ANOVAs were subsequently performed on congruent trials [*F*_(2, 60)_ = 1.34, *p* = 0.269, $\eta_{p}^{2}$ = 0.043] and incongruent trials [*F*_(2, 60)_ = 0.52, *p* = 0.595, $\eta_{p}^{2}$ = 0.017]. In addition, if only participants who were correct on the 2AFC task at 80% or higher (*N* = 9; no participants reached 100% accuracy), there were no meaningful differences in the results [The face type by congruency interaction: *F*_(2, 16)_ = 1.02, *p* = 0.383, $\eta_{p}^{2}$ = 0.113].

### Discussion

In Experiment 3, despite clear recognition of facial features that are indicative of high and low agreeableness, imitation performance was no different compared to the presentation of trait neutral faces. Further, Bayesian analyses show that across all the effects estimated, the null effect is between 2 and 5 times more likely than the experimental effect. Thus, these data suggest imitative tendencies are indifferent to invariant facial cues to agreeableness. This interpretation is limited by the nature of the experimental design used. On trait representative trials, participants saw both high and low agreeable faces within seconds of performing the imitation task. The purpose of pairing faces was to make the distinction between trait diagnostic features as salient as possible. Pairing of faces in this manner, however, makes the influence of positive and negative signals difficult to separate. That is, high agreeable trials could be contaminated by the influence of low agreeable faces and vice versa. To remove potential contamination, Experiment 4 clearly separates the influence of high and low trait signals on imitation.

## Experiment 4

### Introduction

To clearly separate the influence high and low agreeableness on imitation, four changes were made. First, faces were presented singularly instead of in pairs (Figure 1C). Second, the neutral condition was removed, thus leaving only the distinction of high and low agreeable. Third, a blocked design was used, which ensured that in each block participants saw facial cues that signaled only high or low agreeableness. Fourth, we increased our sample size to be as close to 50 as possible in order to increase sensitivity of our tests.

### Methods

#### Participants

Fifty-two Bangor University students participated (33 female, 19 male; *M*_age_ = 19.67 years, *SD* = 2.37). No participants were more than 3SD from the group mean of accuracy or mean RT for congruent or incongruent trials. All provided written informed consent, were right-handed and had normal or corrected-to-normal vision. Compensation for their time was awarded by way of course credit. The Research Ethics and Governance Committee of the School of Psychology at Bangor University granted ethical approval.

#### Task and stimuli

##### Face evaluation task

The face evaluation task was based on prior work (Kramer and Ward, 2010). Two composite images of faces were used: pair one from Experiment 1. Participants were presented with the two composite faces and asked to choose which best represented each of the statements from the 10 items that were relevant to agreeableness on the International Personality Item Pool (IPIP; Goldberg, 1999; found online here: http://ipip.ori.org/MiniIPIPKey.htm).

##### Automatic imitation task

The automatic imitation task was the same as in Experiment 1, except for three changes (Figure 1C). First, participants saw only one example of a high and a low agreeable face and these faces were only ever presented singularly and in blocks. Second, there were no catch trials, as participants saw the same face on every trial within a block. Third, the duration of each ISI was changed to 800, 1200, or 1600 as other studies show robust congruency effects using different ISIs (Brass et al., 2000; Wang et al., 2011; Cook and Bird, 2012).

#### Design and procedure

A 2 × 2 factorial design was used, with factors of congruency (congruent, incongruent) and face type (high agreeable, low agreeable). Participants first completed a 12-trial practice, before the main automatic imitation task. The main task comprised 120 trials divided into two blocks of 60. In different blocks, participants saw the high agreeable composite or the low agreeable composite. Within each block there were 30 congruent and 30 incongruent trials, which produced 30 trials per condition. The order of high agreeable and low agreeable blocks was counterbalanced across participants. The presentation of the target hand image was pseudorandomised so that no same image could appear more than four times consecutively. Following the imitation task, participants completed the face evaluation task. The protocol for this task was the same as for the 2AFC task in Experiment 1.

#### Data analysis

##### Face evaluation

The percentage of correct responses from the total of 10 trials was calculated. This was compared to the percentage that participants would be expected to get if they were performing at chance (i.e., at 50% as it was a 2AFC task).

##### Automatic imitation

Data were processed the same way as in previous experiments. A 2 (face type: low, high) × 2 (congruency: congruent, incongruent) ANOVA was performed on RT as well as estimation of mean differences on CE, congruent RTs, and incongruent RTs between high and low agreeable faces. The same analysis was also performed for those participants who were 100% accurate on the 2AFC face evaluation task.

A sensitivity analysis performed in G*Power showed that using a one-tailed paired *t*-test (e.g., High > Low Agreeable) with a correlation between conditions of 0.5, a sample of 52 we would have 80% power to detect a Cohen's d of 0.35. Further, a sample of 52 would provide over 80% power to detect an interaction between face-type and congruency of partial η^2^ = 0.14.

### Results

#### Face evaluation

Results from the face evaluation task (see Figure 2D) again showed that participants could accurately judge trait information from the static, invariant features with average discrimination accuracy at 76.92% (CI = [69.65, 84.20]; Cohen's d_*z*_ = 1.00) and chance performance at 50%.

#### Automatic imitation

Prior to analysis, trials were removed if participants gave an incorrect response (4.45%), lifted their finger from the “n” or the “m” key during the ISI (0.1%), or took longer than 2000 ms to respond (0.27%).

Mean average RT and standard error of the mean for congruent and incongruent conditions, as well as the CE are reported in Supplementary Table 1. In Table 3 and Figure 5B, estimation information on key contrasts are reported and illustrated. Our key contrasts involve comparing performance on the imitation tasks across high and low agreeable face presentation. Differences between high agreeable compared to low agreeable faces were calculated for the CE −7.30 (CI = [−20.29, 5.70]) with effect size d_z_ = −0.15 and Bayes Factor BF_01_ = 3.64, the congruent condition 12.14 (CI = [−1.98, 26.26]) with effect size d_z_ = 0.24 and Bayes Factor BF_01_ = 1.66 and the incongruent condition 4.84 (CI = [−11.15, 20.84]) with effect size d_z_ = 0.08 and Bayes Factor BF_01_ = 5.55. We also entered the RT data into a 2 face-type × 2 congruency ANOVA, and report the face type by congruency interaction [*F*_(1, 51)_ = 1.27, *p* = 0.265, $\eta_{p}^{2}$ = 0.024]. Additionally, if only those participants who were 100% correct on the face evaluation task were included in the analysis (*N* = 19), there were no meaningful differences in the pattern of results [The face type by congruency interaction: *F*_(1, 18)_ = 0.11, *p* = 0.745, $\eta_{p}^{2}$ = 0.006].

#### Meta-analysis of experiments 3 and 4

**Figure 6:**
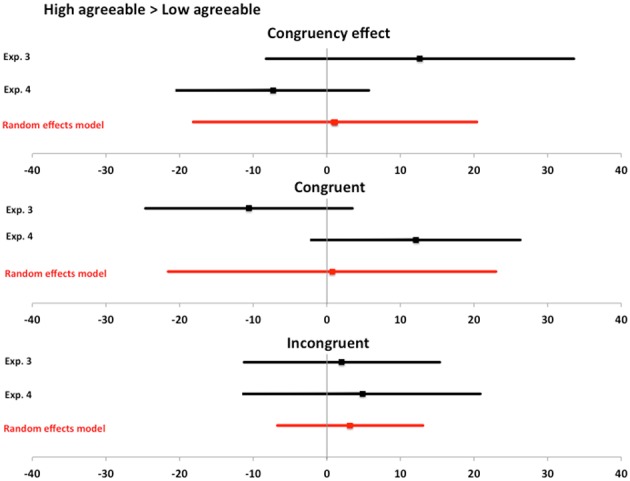
**Meta-analysis for agreeableness data**. Meta-analysis for agreeableness data. Bars represent point estimates and 95% confidence intervals for the effect of interest from each experiment in the meta-analysis, as well as the combined random effects model. For each effect of interest, we report meta-analytical data on the congruency effect, as well as congruent and incongruent conditions. Bars represent 95% confidence intervals and all overlap with zero. Thus, meta-analytical data from Experiments 3 and 4 support the interpretation that the perception of facial features that signal agreeableness show little to no impact on automatic imitation.

We use the same meta-analysis approach as before when analysing the effects of facial expressions (ESCI, Cumming, 2012). Since no prior studies have investigated the impact of facial signals to agreeableness on automatic imitation, we only include Experiments 3 and 4 in the meta-analysis. We compare the difference between high and low agreeable conditions on the congruency effect, as well as congruent and incongruent conditions. We illustrate the results of the meta-analysis in Figure 6 using forest plots. The random effects model shows that our best estimate of the effect of high compared to low agreeableness for congruency effect, as well as congruent and incongruent conditions, is not different from zero (no effect).

### Discussion

Despite accurate recognition of facial features that signal agreeableness, automatic imitation was indifferent to the presentation of a high agreeable compared to a low agreeable face. Even with a clear separation of high and low agreeable faces and increased sensitivity to detect smaller effect sizes, we found no influence on automatic imitation of facial cues that signal stable trait characteristics. Further, Bayesian analyses show that for the CE and incongruent condition, the null effect is between 3 and 5 times more likely than the experimental effect, whereas for the congruent condition the findings are indeterminate and neither support the null nor the experimental effect. Finally, when combining the results of Experiment 3 and 4 in a meta-analysis, we find no evidence that invariant features of an individual's face, which signal agreeableness, modulate imitative tendencies.

## General discussion

Imitation and facial signals underpin and guide social interactions (Haxby et al., 2000; Chartrand and Van Baaren, 2009), but little is known regarding the relationship between mechanisms for face perception and imitation. The current study provides evidence that automatic imitation is modulated by facial cues that signal prosocial, affiliative emotional states more than enduring personality traits. Moreover, the impact of prosocial state signals was dissociable from antisocial and neutral signals. These findings show that facial signals that convey “in the moment” prosocial information are an input to the systems that guide automatic imitation of hand actions.

### Emotional facial expressions and imitation of hand actions

Prior research has suggested that facial imitation during expression detection contributes toward understanding another's emotional state (Niedenthal et al., 2001; Goldman and Sripada, 2005; Wood et al., 2016). The current study extends understanding of the links between facial signals and imitation, by providing further evidence that the perception of emotional states influences inherently non-emotional imitative actions (Rauchbauer et al., 2015). Therefore, we show that facial expressions are an input signal to the control of imitation that extends beyond facial imitation. Although prior work has shown that positively and negatively valenced cues can influence imitation depending on the context (Chartrand and Lakin, 2013), current evidence provides stronger support for a prosocial bias when facial expressions are perceived. Considered together with prior findings, evidence suggests that the perception of smiling regulates multiple forms of imitation, each of which may serve different functions. First, during expression detection, facial muscles are engaged that would be used to produce the expression and contribute to understanding emotional states (Goldman and Sripada, 2005). Second, detection of a smile also impacts imitation of actions that involve distal muscle groups. The latter form of imitation is likely to contribute to facilitating interactions between individuals, such as building rapport and affiliation (Chartrand and Van Baaren, 2009; van Baaren et al., 2009).

From an estimation perspective (Cumming, 2012), our current best estimate of the size of the influence that a smile can have on imitation is considerably smaller than initially reported by Rauchbauer et al. (2015). Depending on the type of effect estimated, the effects reported in the current study are half to 1/3 as large as those reported previously (Cohen's d_z_ 0.37 vs. 0.69 and $\eta_{p}^{2}$ = 0.13 vs. 0.33). There are many design differences that could account for differences in the effects observed, which future research should explore. For instance, Rauchbauer et al. (2015) crossed an expression manipulation with a group bias manipulation based on race (ingroup race vs. outgroup race), which makes the sequence of stimuli more variable in terms of social information. In addition, given that we should expect effect sizes to vary to some degree across studies (Cumming, 2012), it will take many more experiments and meta-analytic approaches to provide a more robust estimation of the true population effect size.

### The importance of reporting null results

We found no evidence that recognizing invariant facial features that are associated with agreeableness has any impact on imitation and Bayesian analyses consistently favor the null hypothesis over the experimental hypothesis. Although null results are not straightforward to interpret, following suggestions that publishing null results is important in order to avoid the “file drawer” problem (Rosenthal, 1979), we hope that these findings can foster a more informed set of future experiments.

The null result could reflect that there is no influence of cues associated with agreeableness on imitation. Alternatively, the null result could reflect that the influence we are trying to detect is subtle. In our most sensitive test (Experiment 4), we can rule out an effect size larger than a Cohen's d_z_ of 0.35 with reasonable confidence (80%). However, the effect size could be smaller than we are able to confidently reject. Relatedly, affiliative motivations have been cited as a key driving force for regulating imitation (Chartrand and Lakin, 2013) and the effect of trait signal may have been too subtle to influence affiliative motivations. Nonetheless, our current best estimate favors the null effect (no difference) as well as a much smaller effect than the influence on imitation of perceiving a smile. From an ecological validity standpoint, if facial expressions are a stronger visual cue than invariant trait features in day-to-day life, expressions may indeed impact automatic imitation more than stable trait cues. Thus, the observed differences between invariant face cues and emotional expressions could be because expressions provide a more intense social signal albeit one that reflects authentic social exchanges. Future work could address this question further by showing less intense versions of emotional expressions to investigate whether there is a relationship between the intensity of the facial signal and automatic imitation.

### Limitations and future directions

A limitation of the current study and future line of research would be to investigate the influence of facial cues to other stable traits. In the current study we investigated the influence of facial cues to trait agreeableness. However, trustworthiness and dominance (Todorov et al., 2008, 2015), as well as extraversion (Penton-Voak et al., 2006; Kramer and Ward, 2010) are also perceived from invariant facial features. These traits could also be important for modulation of imitation behavior and as such would provide interesting possibilities for further research.

Whilst it is becoming clear that a range of social factors impact imitation (Heyes, 2011; Chartrand and Lakin, 2013), the mechanisms by which they operate are largely unknown. For example, a limitation of the current study, and others prior, is that it is unclear if the influence of social cues on imitation is anchored to the self, the target or a combination of both. If the mechanism were anchored to the self, through an elevation in mood for example (van Baaren et al., 2006) or a general desire to affiliate for instance, imitation would increase with their interaction partner as well as with anyone else with whom they might interact. By contrast, if the mechanism were anchored to the social target in an attempt to build social connections with a particular individual, imitation would not generalize to other interaction partners. Future research might try to disentangle these possible factors, for example including a mood questionnaire, to further understand the mechanisms by which imitation is controlled.

Finally, inclusion of baseline trials in the automatic imitation task (Rauchbauer et al., 2016), where participants respond to a number cue when the observed hand remains still could have clarified whether response times were generally speeded up or slowed down in response to the presentation of emotionally expressive faces. This could elucidate whether expressive faces influenced basic reaction times or whether the influence of expressions is specific to an imitative response. In line with this, a non-social interference task would also provide insight into whether the results observed are specific to an imitative response or a more general interference response. These options remain interesting questions for future research.

## Author contributions

EB contributed to stimuli, task design, testing, analysis, and writing of this manuscript. RW contributed to stimuli, task design, and writing of this manuscript. RR contributed to stimuli, task design, analysis, and writing of this manuscript.

### Conflict of interest statement

The authors declare that the research was conducted in the absence of any commercial or financial relationships that could be construed as a potential conflict of interest.

## Footnotes

^1^Cohen's d is a measure of effect size with values of 0.2, 0.5, and 0.8 generally considered small, medium, and large effects, respectively (Cohen, 1992).

^2^JASP Team (2016). JASP (Version 0.7.5.5) [Computer software].

^3^Partial eta squared (Partial η^2^) is a measure of effect size used in ANOVA with benchmark values of 0.01, 0.06, and 0.14 considered small, medium, and large effect sizes, respectively (Cohen, 1988).

^4^Whilst this manuscript was under review an additional paper was published (Rauchbauer et al., 2016). Rauchbauer and colleagues used a lab-based measure of imitation and across three experiments found that imitation was greater for happy in-group faces than happy out-group faces, and was greater for angry out-group compared to angry in-group faces. In both cases, increased imitation was the result of faster RTs on congruent trials. Due to the timing, Rauchbauer et al. (2016) did not inform our introduction or task design however the authors kindly provided their data to use in our meta-analysis for completeness of reporting previous effects. In order to compare Rauchbauer et al. (2016) findings to previous research (Table 1) we have included information in Supplementary Table 2.
