# Supplementary material for: The Influence of Facial Signals on the Automatic Imitation of Hand Actions
Source: Front Psychol. 2016 Oct 26;7:1653. doi: 10.3389/fpsyg.2016.01653 (PMC5080362; doi:10.3389/fpsyg.2016.01653)
Supplement: Supplementary file 1 [file DataSheet1.docx]

***Supplementary Material***

**The influence of facial signals on the automatic imitation of hand actions**

**Emily E. Butler, Robert Ward & Richard Ramsey**

Wales Institute for Cognitive Neuroscience, School of Psychology, Bangor University, Adeilad Brigantia, Penrallt road, Bangor, Gwynedd, LL57 2AS Wales

Corresponding authors:

Emily Butler & Richard Ramsey

Wales Institute for Cognitive Neuroscience, School of Psychology, Bangor University, Adeilad Brigantia, Bangor, Gwynedd, LL57 2AS Wales

**Supplementary Table 1 – Mean and standard error across Experiments 1 to 4**

|  | **Experiment 1** | | | | | | | | | |
| --- | --- | --- | --- | --- | --- | --- | --- | --- | --- | --- |
|  | **Happy** | | | **Angry** | | | **Neutral** | |  | |
|  | *Mean* | | *SEM* | *Mean* | *Mean* | | *SEM* | *Mean* |  |  |
| CE | 85.55 | | 8.49 | 93.51 | 85.55 | | 8.49 | 93.51 |  |  |
| Congruent | 495.30 | | 13.16 | 482.14 | 495.30 | | 13.16 | 482.14 |  |  |
| Incongruent | 580.86 | | 15.20 | 575.65 | 580.86 | | 15.20 | 575.65 |  |  |
|  |  | | |  | | |  | |  | |
|  | **Experiment 2** | | | | | | | | | |
|  | **Happy** | | | **Angry** | | | **Neutral** | | **Salient Neutral** | |
|  | *Mean* | *SEM* | | *Mean* | | *SEM* | *Mean* | *SEM* | *Mean* | *SEM* |
| CE | 122.35 | 11.65 | | 102.18 | | 10.54 | 122.35 | 11.65 | 102.18 | 10.54 |
| Congruent | 483.69 | 12.59 | | 489.13 | | 12.26 | 483.69 | 12.59 | 489.13 | 12.26 |
| Incongruent | 606.03 | 20.50 | | 591.32 | | 18.03 | 606.03 | 20.50 | 591.32 | 18.03 |
|  |  | | |  | | |  | |  | |
|  | **Experiment 3** | | | | | | | | | |
|  | **High**  **Agreeable** | | | **Low Agreeable** | | | **Neutral** | |  | |
|  | *Mean* | *SEM* | | *Mean* | | *Mean* | *SEM* | *Mean* |  |  |
| CE | 116.99 | 8.99 | | 104.36 | | 116.99 | 8.99 | 104.36 |  |  |
| Congruent | 495.83 | 14.85 | | 506.44 | | 495.83 | 14.85 | 506.44 |  |  |
| Incongruent | 612.82 | 16.73 | | 610.80 | | 612.82 | 16.73 | 610.80 |  |  |
|  |  | | |  | | |  | |  | |
|  | **Experiment 4** | | | | | | | | | |
|  | **High**  **Agreeable** | | | **Low Agreeable** | | |  | |  | |
|  | *Mean* | *SEM* | | *Mean* | | *SEM* |  |  |  |  |
| CE | 82.55 | 6.89 | | 82.55 | | 6.89 |  |  |  |  |
| Congruent | 518.41 | 12.52 | | 518.41 | | 12.52 |  |  |  |  |
| Incongruent | 600.96 | 15.62 | | 600.96 | | 15.62 |  |  |  |  |
|  |  |  | |  | |  |  |  |  |  |

**Supplementary Table 2 -** **Results from Rauchbauer et al. (2016)**

| **Study / Sample size /**  **Manipulation** | **Contrast** | **Mean difference (ms)** | | **95% Confidence intervals** | **Cohen’s d_z_** | **Bayes factor BF_01_** |
| --- | --- | --- | --- | --- | --- | --- |
|  |  |  |  |  |  |  |
| **Rauchbauer et al., (2016)** | Happy > Angry |  |  |  |  |  |
| Exp. 1 N = 62 |  | CE | 3.29 | [-3.91, 10.48] | 0.12 | 4.83 |
|  |  | Congruent | 1.26 | [-3.14, 5.65] | 0.07 | 6.15 |
|  |  | Incongruent | 4.55 | [-1.10, 10.19] | 0.2 | 2.13 |
|  |  |  |  |  |  |  |
| Exp. 2 N = 61 |  | CE | -4.55 | [-11.33, 2.23] | -0.17 | 3.05 |
|  |  | Congruent | 1.71 | [-1.96, 5.38] | 0.12 | 4.73 |
|  |  | Incongruent | -2.84 | [-8.25, 2.57] | -0.13 | 4.22 |
|  |  |  |  |  |  |  |
| Exp. 3 N = 58 |  | CE | 1.18 | [-6.57, 8.94] | 0.04 | 6.66 |
|  |  | Congruent | 4.15 | [-3.73, 12.02] | 0.14 | 4.11 |
|  |  | Incongruent | 5.33 | [-2.17, 12.83] | 0.19 | 2.69 |
|  |  |  |  |  |  |  |
